# The future of medicines use and access research: using the *Journal of Pharmaceutical Policy and Practice* as a platform for change

**DOI:** 10.1186/2052-3211-7-8

**Published:** 2014-07-08

**Authors:** Zaheer-Ud-Din Babar, Andy Gray, Ayyaz Kiani, Sabine Vogler, Peri Ballantyne, Shane Scahill

**Affiliations:** 1 School of Pharmacy, University of Auckland, Private Mail bag 92019, Auckland, New Zealand; 2 Division of Pharmacology, Discipline of Pharmaceutical Sciences, University of KwaZulu, Natal PBag 7, Congella 4013, South Africa; 3 Devnet Consultants, Islamabad, Pakistan; 4 Gesundheit Österreich GmbH, Stubenring 6, 1010 Wien, Austria; 5 Trent University, Peterborough, ON, Canada; 6 School of Management, Massey University, Auckland, New Zealand

## 

Scientific journals are most often used to disseminate the end products of research, but also have an important role as instruments of change. One year after the launch of the *Journal of Pharmaceutical Policy and Practice*, the focus of articles published has been on pharmaceutical health systems research, including contemporary issues related to medicines management, socio-behavioral aspects of pharmacy and macro pharmaceutical issues. Our most cited articles ranged from those on generic medicines in Jordan [1], antibiotics sensitivity, usage and access in India and Namibia [2,3], to a review of national medicines policies around the globe [4]. At this point the Journal has successfully provided a forum to publish within its specified themes. However, given the technological and social changes in health, medicines and public policy, we are keen to promote the *Journal of Pharmaceutical Policy and Practice* as a platform for change, in order to advance specific agendas. We would argue that this change agenda is underpinned by the following issues:

## Global demographic change: growing health disparities

Global demographic change encompasses far more than declining fertility and an aging population. Social and human capital are far more mobile than they once were. Immigration has resulted in multicultural populations in most developed countries [5]. For example, in United States alone, 321 different languages are spoken. By 2050, what are currently regarded as racial and ethnic minorities will constitute 50% of the total population of the US [6]. Health disparities among these populations are of particular concern [7]. What is of most interest to readers of this journal is how these demographic changes will impact on medicines use, health, disease and public policy. A proactive research agenda focusing on these issues is required.

## Changes in healthcare and community pharmacy practice

Worldwide, a community pharmacy is generally the most common point at which the public accesses medicines, as well as health advice. However, in many countries, community pharmacy is seen as having interests that are oppositional to public health and motivated purely by profit. As a result, community pharmacy as a sector is often marginalised from the health and social care system. It is seen as a rather insular profession, busy with its own concerns, and not engaged with debates and decisions in the wider world of health policy [8]. Medicines use issues at the points of transition between ambulatory and institutional healthcare are also fertile areas for investigation and innovation.

It has been argued that pharmacy practice research has a lower profile than research undertaken by other healthcare professionals [9]. This is especially surprising at a time when some governments are advocating greater integration of pharmacy into mainstream healthcare and also facing increasing demands for access to expensive medicines [10]. Although pharmacy practice research has recently attracted large grants, and some robust evidence is emerging, there remains a paucity of knowledge on the quality of services delivered by pharmacists and a lack of evidence in terms of patient outcomes and value for money. Indeed, what evidence does exist is dominated by data from developed country settings [11].

Successful implementation of pharmacy practice research cannot be achieved simply by information dissemination, training, regulation and compliance with guidelines. Just because a programme or service has been shown to demonstrate positive outcomes for patients does not mean that it will be easily implemented in practice [12,13]. For example, a recent study [14] of the community pharmacy Medicine Use Review (MUR) services in England showed that the success of this new patient–pharmacist model of interaction was dependent on patients’ understanding of the pharmacists’ role, the perceived hierarchy and position held by general practitioners, and experience of what actually occurs during the MUR interaction [14]. We need to challenge our practices and beliefs if things are to change and the future research agenda on pharmacy service models has to focus on these issues [15]. In addition, pharmacy practice research has to be seen as linked to overall pharmaceutical policy, including pharmaceutical pricing and reimbursement strategies and demand-side measures to, for instance, enhance generic uptake and/or a more rational use of medicines.

## Technology is rapidly changing

There is an increasing use of technology in the delivery and management of healthcare. There is use of automation and robotics at hospital and community pharmacies, electronic prescribing, e- communication, use of robotics, pharmacists’ access to integrated patient records- all are changing at a very fast pace and are directly related to how patients and consumers are accessing and using medicines [8]. Social media is on the rise and there is no reason to suggest that this will be any less the case for those engaged with the pharmacy sector

## What could be on the new research horizon?

In the context of the trends outlined here, what will the healthcare sector look like in 20–30 years? What might medicines use and access look like? It is likely there will be many changes and the way we think, act and formulate research questions will be different. Might many of the current questions we are asking be irrelevant? Might new professional alliances be formed that transform the effectiveness of how the public is using medicines? To enable such a shift, there will be a need for a significant rethink of the models of care through which medicines are delivered. New approaches to delivering patient services will emerge [8].

Researchers can no longer expect to secure funding for projects solely focused on pharmacy practice [16]. In order to maximize the impact of their work pharmacy practice researchers will need to include physicians and nurses at the very least and preferably other health professionals [16]. Moreover, pharmacy practice research teams have to involve researchers with different academic backgrounds such as sociologists, economists, and epidemiologists in-order to have their research more widely recognized and accepted. The healthcare system of the future demands a connected, transdisciplinary and collaborative approach, and the same applies to pharmaceutical policy and practice research. The *Journal of Pharmaceutical Policy and Practice* stands ready to not only publish that research, but to promote that agenda, and to ask those tough and relevant questions.
